# *Bonheur en boule*: an adapted group-based physical activity program for youth with disabilities

**DOI:** 10.3389/fspor.2025.1580697

**Published:** 2025-07-31

**Authors:** Jason D’Amours, Stéphanie Girard, Paule Miquelon, Pierre-Luc Veillette

**Affiliations:** ^1^ Department of Psychology, Université du Québec à Trois-Rivières, Trois-Rivières, QC, Canada; ^2^ Department of Human Kinetics, Université du Québec à Trois-Rivières, Trois-Rivières, QC, Canada

**Keywords:** physical literacy, self-determination theory, disabilities, adapted physical activity, youth, program, intervention

## Abstract

**Introduction:**

Youth with disabilities face significant barriers to physical activity (PA) participation, despite its documented benefits across cognitive, social, affective, and physical domains. Physical literacy (PL) and self-determination theory (SDT) offer complementary frameworks for designing adapted PA programs that foster autonomy, competence, and relatedness. However, limited research integrates both frameworks in adapted PA programs. This study evaluates the impact of *Bonheur en boule* (BEB), an adapted group-based PA program, on basic psychological needs, global self-esteem, and PA intentions of youth with disabilities while also assessing parental satisfaction.

**Methods:**

A mixed-methods approach was used. Eleven youth (*M*age = 13.27, *SD* = 5.42) with various disabilities participated in the *BEB* program, consisting of two 15-week sessions of adapted Dek Hockey (Ball hockey). Quantitative measures assessed participants' basic psychological needs (autonomy, competence, relatedness), global self-esteem, and PA intentions at three time points. Non-parametric tests (Friedman and Wilcoxon signed-rank) were used for statistical analyses. Parent satisfaction was evaluated through semi-structured interviews, analyzed using content analysis.

**Results:**

Significant improvements were observed across all three basic psychological needs and global self-esteem. Competence and autonomy satisfaction increased notably between the first and second time points (*p* < 0.01), while global self-esteem improved later in the program (*p* < 0.01). All participants (*n* = 11) expressed intentions to continue both PA and the program. Parental feedback highlighted positive changes in children's cognitive (e.g., attention, problem-solving), social (e.g., friendships, teamwork), affective (e.g., emotional regulation, confidence), and physical (e.g., motor skills, endurance) development. Parents also valued the program's inclusive approach and flexibility.

**Discussion:**

Findings suggest that an SDT and PL based PA program can foster satisfaction of basic psychological needs leading to self-determination, increase global self-esteem and support PA engagement among youth with disabilities. The program's structure, emphasizing autonomy, competence, and social connections, contributed to participant development. Parents' strong appreciation underscores the program's perceived effectiveness. However, challenges such as small sample size and the lack of a control group limit generalizability.

**Conclusion:**

*BEB* demonstrates the feasibility of an adapted PA program rooted in SDT and PL principles, showing promising outcomes for youth with disabilities. Future research should explore long-term behavioral impacts and broader implementation strategies.

## Introduction

Physical activity (PA) programs are a promising avenue for youth with disabilities to obtain benefits in various areas: social (e.g., relationships, communication), physical (e.g., motor skills, cardiometabolic health), cognitive (e.g., executive functions, attention), and affective (e.g., well-being, self-esteem) (1). Moreover, active young people with disabilities demonstrate higher self-esteem (individual's perception of their self-worth) (2, 3) than inactive individuals, suggesting that physical activity is a foundation for life-long PA habits and a healthy lifestyle as well as an indicator of well-being (4, 5). In line with this observation, research suggests that participation in organized youth sports or PA correlates with increased leisure-time PA in adulthood (6–8). Promoting participation in organized sports, such as adapted PA programs during childhood and adolescence, thus emerges as a promising strategy for nurturing both PA and self-esteem among young people with disabilities. A major obstacle, however, is the absence of appropriate programs tailored to youth with disabilities (9). Furthermore, parents, who play a fundamental role in facilitating their children's participation, often face numerous challenges, including time constraints, misperceptions about their children's abilities and a lack of appropriate resources (10–12). As well, they often express concerns regarding quality of supervision, inadequate facilities and their children's safety (10, 13). Additional obstacles, such as limited social support, fear of injury or negative experiences and logistical issues like transportation, further fuel their reluctance to encourage PA participation (14–16). In consequence, parents often act as key moderators, influencing the motivation for and frequency of their child's participation in PA, which underscores the critical importance of their satisfaction with such programs.

In recent years, physical literacy (PL) has been recognized as a foundational element in guiding actions to encourage youth's participation in PA (17, 18). PL is defined as “the motivation, confidence, physical competence, knowledge, and understanding to value and take responsibility for engagement in PA for life” (19). This comprehensive definition highlights the multifaceted nature of PL, which, as research suggests, nurtures not only physical competence but also cognitive, behavioural, and affective development (20). Moreover, when PL is integrated into a PA program, its impact broadens to include a crucial social component that fosters collaboration and builds meaningful relationships. Indeed, group PA serves as a rich social experience, generating a sense of peer acceptance and creating valuable opportunities for friendship, which are especially critical for youth with disabilities, who often experience difficulties forming connections and typically have fewer friends (21–23). The development of PL is equally, or even more, important for young people with disabilities as PL encourages the adoption of habits that promote regular PA, which, in turn, supports the social aspect of these children's development (24).

Despite the emergence of PL-based programs in recent years, significant challenges remain regarding the development of effective PA programs for youth with disabilities. These programs must be responsive and carefully adapted to the specific needs of participants in order to achieve positive outcomes (21, 25, 26). To this end, self-determination theory (SDT) (27, 28) offers a comprehensive framework that emphasizes the importance of satisfying basic psychological needs, which then promotes motivation among youth with disabilities (29).

According to SDT, the satisfaction of three basic psychological needs—autonomy, competence and relatedness—motivates individuals across various ages and contexts to engage in an activity. When these needs are met, individuals experience intrinsic motivation and well-being and are encouraged to persevere (30). Autonomy refers to the ability to make choices based on personal values, interests and personality. Adapted PA programs can support this need by offering participants choices and opportunities to experiment on their own. Relatedness refers to the development of a sense of belonging, connection and social support, which helps foster positive relationships with others. This need can be fulfilled when participants in a PA program feel they belong to a group that includes them in its different activities and accepts them for who they are. Additionally, offering every participant the same opportunities to develop friendships should reinforces this sense of inclusion within the context of an adapted PA program. Competence refers to the sense of achievement that comes from learning and improving skills and performing tasks successfully. According to SDT, the structure of an activity should enable individuals to learn and to develop a sense of competence. Thus, offering clear explanations of drills and planning tasks using visual support based on participants' abilities and capacities, while offering realistic challenges, is recommended in this regard. As well, supporting improvement and recognizing effort can further promote participants' sense of skill development and mastery. Taken together, to foster the intrinsic motivation of young people with disabilities, therefore, PA programs should be designed to satisfy the basic psychological needs of participants to promote greater engagement and perseverance towards PA (28, 30, 31).

**Figure 1:**
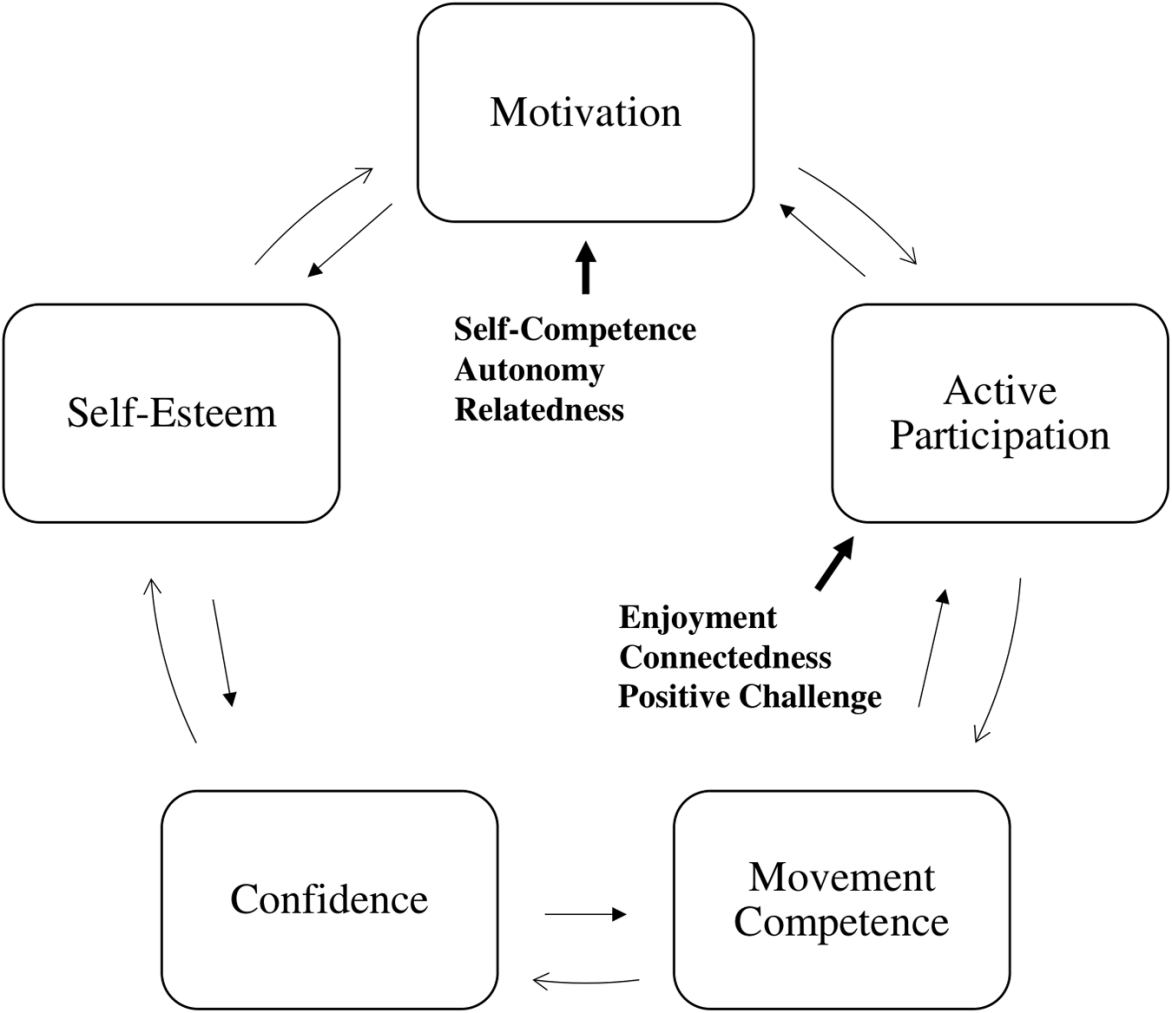
Physical literacy cycle [adapted from Cairney et al. (32); Girard et al. (17); Jefferies et al. (33); Stuckey et al. (34)].

SDT concepts have recently been integrated into the PL cycle (17, 32–34), however, few studies employ PL and SDT as a framework when designing PA programs for youth with disabilities (35–37). As Figure 1 shows, the development of PL encourages participation in PA, which leads to the development of PL (38). According to the PL cycle, motivating children by meeting their basic psychological needs is conducive to their active participation, which will then develop their movement competence and, in parallel, their confidence and motivation (32–34). This increased confidence, moreover, helps participants develop higher self-esteem insofar as they feel better able to participate and consequently more motivated to engage in PA. A sense of confidence helps develop the child's motor competence, while experiencing positive challenges, fun and connection through participation in PA fuels the motivation to be physically active.

Overall, motivation and self-esteem are essential for active participation in PA. When basic psychological needs are met, the result is a positive environment that promotes enjoyment and social connectedness. Higher self-esteem, in turn, builds confidence and a sense of competence, which further increases the motivation to participate. Thus, the integration of SDT within the PL cycle represents a relevant framework for designing adapted PA programs that enhance participation among youth with disabilities.

While several programs have been developed in recent years for youth with disabilities, significant challenges remain when it comes to evaluating their scientific value and effectiveness. Key limitations include a lack of evaluation methods and the obstacles to implementing such programs, making it more difficult to capture the full range of outcomes, from physical improvements to psychological and social benefits.

Indeed, these programs often struggle to address the wide range of disabilities, including cognitive, sensory and physical impairments, and may lack sufficient adaptations to ensure equitable participation and inclusivity (39). This limitation restricts their capacity to present effective strategies, create inclusive group settings and provide specific adaptations that meet the diverse needs of the disability community. Chen et al. (40), for example, highlight the difficulty of designing a soccer program to accommodate participants with varying levels of ability, experience and types of disabilities. This diversity complicates the creation of universally effective programs. Choi (41), on the other hand, notes that homogeneous group settings fail to capture the diversity of the broader population of youth with disabilities, limiting the generalizability of program outcomes. The necessity for more inclusive settings that address the unique needs of participants is critical. However, the implementation of such programs faces persistent obstacles, including limited funding, inaccessible facilities and a lack of qualified staff (42–44).

On a methodological level, Perić et al. (45) and Hsu et al. (46) stress that when sample groups consist of participants with similar ages or backgrounds, it becomes difficult to generalize the results to a broader population, which limits the external validity of studies of such programs. A further limitation is the reliance on parents to complete questionnaires or assessments. According to Morales (47), parental involvement in data collection can introduce bias, particularly since parents may under- or overestimate their child's progress. Another pressing issue is the long-term sustainability of these programs, which often lack scientific evidence, suggesting uncertainty and the challenges many face to maintain their effectiveness over time (48). Finally, little information is available as regards fidelity of program implementation (39). Gaining a clear understanding of program fidelity is therefore essential to determine the true impact of these interventions and ensure their effective replication or scalability.

To move forward, future interventions should prioritize a replicable and more driven universal design, focus on comprehensive staff training and foster collaboration among researchers, practitioners and families. Expanding research into adaptive program models and inclusive frameworks is therefore essential to ensure these programs meet the needs of all participants, ultimately fostering greater access to PA opportunities. Given these challenges and methodological limitations, the program presented and used in the current study attempts to address some of these limitations, particularly in terms of specific adaptations, replicable framework designs and clearer implementation strategies.

More explicitly, despite the theoretical relevance of PL and SDT in promoting PA among youth with disabilities, few to no program specifically integrate both frameworks into their design. Moreover, the existing literature often overlooks several implementation factors, such as the adaptability details of programs in community settings, the psychological outcomes for participants and the role of the parents regarding program participation.

To address these gaps, this study aims to evaluate the effectiveness of a recently adapted PA program, *Bonheur en boule* (*BEB*), which was designed to develop the PL of youth with various disabilities. Fostering inclusivity through a heterogeneous group dynamic within a community setting, the *BEB* program aligns with SDT postulates (see Supplementary file S1). Specifically, the objectives are as follows: (1) measure changes in participants' basic psychological needs' satisfaction (autonomy, competence and relatedness) and global self-esteem during program participation; (2) measure participants' intention to pursue the program and practice PA afterwards (behavior component of PL); and (3) describe parents' satisfaction with *BEB*.

## Materials and method

### Study design and measures

A mixed method design was used to gather quantitative data on the evolution of measured variables and qualitative data regarding parents' perception of the program. This approach was chosen to gain a nuanced understanding of participants' experiences and outcomes during their time in the program (49). It enabled the integration of quantitative measures to assess changes in participants' basic psychological needs, self-esteem and intention to pursue PA, while qualitative data collection offered insights into parents' satisfaction with the program and enriched the interpretation of quantitative findings. The mixed method design allowed us to identify possible explanations for the presence or absence of changes in the variables measured. By combining both quantitative and qualitative methods, the present study takes a pragmatic perspective, which aims to enhance the validity and comprehensiveness of the methodological approach (50).

### Recruitment

**Figure 2:**
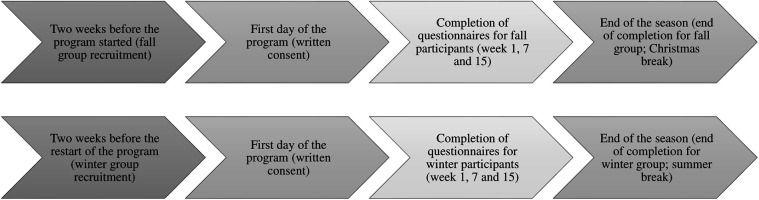
Recruitment timeline.

Prior to recruitment of participants, ethical approval was obtained from the first author's institutional research ethics board [CERPPE-23-19-07.01]. To avoid bias and pressure and promote voluntary participation, the primary instructor, who is also the main author, was withdrawn from the data collection process. As a result, he could not know who participated in the research. Two weeks before start of the program, a research assistant initiated recruitment by posting a video introduction to the project on the main Facebook group page, accessible to all registered participants. Group members were invited to signal their interest by contacting the research assistant via email. The assistant arrived early the first day to distribute consent forms, provide further details and obtain the written consent of all participants and parents. This process was repeated for both the fall and winter seasons (see Figure 2). Parents were recruited using the same Facebook group page, which invited them to an interview on their satisfaction with the program.

### Participants

The study involved youth from two distinct cohorts, representing both the fall and winter seasons. A total of 12 male participants (*M*_age_ = 13.27 years old, *SD* = 5.42) contributed to the experiment; they included seven individuals (*n* = 2 developmental coordination disorder; DCD, *n* = 1 intellectual disability; ID, *n* = 1 Down syndrome; DS, *n* = 1 developmental delay; DD, *n* = 1 DS and ID, *n* = 1 developmental delay; DD, *n* = 1 autism spectrum disorder; ASD and oppositional defiant disorder; ODD) from the fall cohort and four individuals (*n* = 1 DCD, *n* = 1 ID AND DCD, *n* = 1 DD, *n* = 1 ODD) from the winter cohort. All participants included in the study were boys, possibly because of the higher prevalence of certain disabilities (e.g., ASD) and the specific interest towards Dek Hockey (ball hockey) (51). Because one participant was unable to complete the study, analyses were conducted using a total of 11 participants (see Table 1).

**Table 1:** Description of BEB groups.

	Min	Max	*M*	*Md*	*SD*
Autumn (*n* = 7)					
Age	10.00	24.00	16.00	16.00	4.73
Years in *BEB*	1.00	7.00	4.00	3.00	2.38
Winter (*n* = 4)					
Age	7.00	12.00	8.50	7.50	2.38
Years in *BEB*	.00	1.00	.25	.00	.50
Total (*n* = 11)					
Age	7.00	24.00	13.27	12.00	5.42
Years in *BEB*	.00	7.00	2.64	2.00	2.66

Note: Age, years old; Years in *BEB*, number of years of participation in the *BEB* program; *M*, mean; Min, minimum; Max, maximum*; Md*, median; *SD*, standard deviation; Total, autumn and winter groups.

### Intervention—*BEB program*

*Bonheur en boule* (*BEB*) is a PA program adapted to meet the needs of children, adolescents and young adults (aged 5–24) with disabilities (multiple disorders: Down's syndrome, intellectual disability, autism spectrum disorder, and so on). More specifically, it is an adapted Dek Hockey program aimed at meeting the needs for autonomy, competence and relatedness. Training sessions took place every Saturday at the exact same time. The program was divided into two 15-week seasons respectively during fall (September to December) and winter (January to April). Each session lasted 60 min (see Supplementary file S1) and consisted of a free-play period (warm-up), a training period (practice), a game period (play), and an endgame (cool-down). The activity was supervised by six volunteers with a background in intervention (e.g., psychoeducation, psychology, education, etc.). The main goal was to offer youth with disabilities an opportunity to practice sport in way that was safe, fun and enjoyable. See Supplementary file S2 for detailed adaptions through each phase of the program (arrival to end).

#### Eligibility

Participants were eligible if they were five to 24 years old and presented disabilities. The program is inclusive, and many exceptions have been made in the past (e.g., acceptance of a 28-year-old participant). To date, no registration has been refused.

### Procedure and data collection

On the first day of the program, a research assistant arrived 30 min early to guide participants in completing the questionnaire behind closed doors, allowing no contact with the main instructor. Once done, each participant was free to go to the playground where an assigned assistant instructor supervised the free-play period. This process was the same for all questionnaires and took place at season's start (session 1; T1), in mid-season (session 7; T2) and at season's end (session 15; T3) for both cohorts of participants (fall and winter). Each questionnaire included a special three-number code (99-99-99) created by the participants to ensure confidentiality before the main author associate the questionnaires for each measurement time.

As for assessing parents' satisfaction with the program, another research assistant contacted interested parents to determine their preferred time for the interview; they were then sent an email with the appropriate ZOOM link and secret code. During the meeting, participants were asked for permission to record, and the interview's objectives were repeated. After the meeting, parents were asked for additional comments and thanked for their participation. The research assistant typed the verbatims, which were anonymized to prevent identification by the main author.

### Instruments

Each questionnaire included 25 questions, took about 15 min to complete and consisted of five scales (global self-esteem, autonomy need satisfaction, competence need satisfaction, relatedness need satisfaction, and intention to be physically active) validated in French in previous studies. All scales displayed acceptable internal consistencies (*α* ≥ 0.70) (52–55). The Cronbach's (*α*) and Omega's (*ω*) values presented in the next subsections were calculated using the sample in the present study.

To measure global self-esteem, we used the French version of the Self-Esteem Scale (ÉES-10) (55), which includes 10 items (*α* = 0.78; *ω* = 0.79; e.g., *On the whole, I'm satisfied with myself*). Participants answered the questions using a 4-point Likert-type scale ranging from (1) *Totally disagree* to (4) *Totally agree*. To measure satisfaction of the three basic psychological needs, the Psychological Needs Questionnaire, adapted in French by Girard et al. (17), was used. This SDT-based scale has been previously used with similar populations and various types of disabilities in the context of PA (31, 56, 57). This questionnaire contains five items to assess satisfaction of each of the three needs (autonomy, competence, relatedness) for a total of 15 items, measured with a 7-point Likert-type scale ranging from (1) *Strongly disagree* to (7) *Strongly agree.* The competence scale, as used by Standage, Duda and Ntoumanis (58), is based on the Intrinsic Motivation Inventory (59) and includes five items (*α* = 0.75; *ω* = 0.73; e.g., *Since the start of the program, in my Dek Hockey sessions, I feel I'm quite skilled*). The autonomy scale, also developed by Standage, Duda and Ntoumanis (58), contains five items (*α* = 0.64; *ω* = 0.74; e.g., *Since the start of the program, in my Dek Hockey session, I feel I can choose what activity to do*). However, in the present study, only four items were used, as removing one item (i.e., *I engage in physical activity because I want to*) led to a better internal consistency (*α* = 0.54: *α* = 0.64; *w* = 0.66: *ω* = 0.74), thereby enhancing the reliability of the autonomy scale without compromising its conceptual validity. This item was removed because it was potentially misinterpreted by participants, as its phrasing lacked specificity and could lead to different interpretations depending on personal experiences or functional limitations. Finally, to assess relatedness satisfaction, we used the *Échelle du sentiment d'appartenance sociale* (54) consisting of five items (*α* = 0.78; *ω* = 0.77; e.g., *From the start of the program, in my Dek Hockey session, I feel listened to by my peers*).

To measure participants' intention to practice PA in the next three months and pursue the program as part of the PL behavior component targeting engagement in PA, four questions were added to the final version of the questionnaire (T3). All questions were inspired by the French version developed by Boudreau and Godin (52), which measures the intention to practice PA in the next three months using a 7-point Likert-type scale ranging from (1) *Very unlikely* to (7) *Very likely.* The final question specifically targets the participant's intention to re-enroll in the program and was answered by checking *Yes, No* or *I don't know.* It is, however, important to note that intentions to engage in PA were not verified through objective behavioral data.

#### Interviews

The individual semi-directed interview guide was created by the author of the present study and consists of seven questions asking parents about their satisfaction with *BEB* (e.g., *How do you rate your overall experience with the program so far?*) and the benefits they anticipated for their child following participation in the program (e.g., *What aspects of this program did you find most beneficial for your child?*). The interviews were conducted by a research assistant who ensured that participants responded based on their own perceptions and did not seek to guide their answers. The assistant also made sure parents felt comfortable sharing their answers in a respectful context conducive to exchange, and reassured them there were no right or wrong answers by clarifying the objectives at the start of the interview. See Supplementary file S3 for additional details about the interview framework, which has been freely translated from French.

### Analyses

#### Quantitative analyses

Data were analyzed using SPSS statistical software (version 29). After the data were cleaned, descriptive analyses (i.e., means, median and standard deviation) were conducted to describe the sample and display the time-point comparison for basic psychological needs, global self-esteem and intention variables. Because of the small sample size and non-normality of the data (i.e., asymmetricity of distribution around the mean) suggested by kurtosis and skewness analyses, non-parametric tests were performed. The standard Friedman test (60) was used to determine if differences existed among the three time points across the following variables: autonomy, competence, relatedness and global self-esteem. An *a priori* alpha level of.05 was established as the threshold for significance. To control for the increased risk of false positives arising from multiple pairwise comparisons, a *post hoc* Bonferroni correction was applied, resulting in a revised significance level of.017 (60). The Wilcoxon signed-rank test was used because of its suitability for paired, non-parametric data. Effect sizes (r) were also calculated for Wilcoxon signed-rank tests comparisons to enhance the interpretive value of the results. The *post-hoc* Wilcoxon signed-rank test analysis enabled us to measure changes in participants' basic psychological needs and global self-esteem following their participation in the *BEB* adapted PA program and to identify the differences across the three time points (i.e., before the first session; T1, 7th session; T2, 15th session; T3).

#### Qualitative Analyses

Qualitative analyses were conducted using L'Écuyer's (61) content analysis method, which identifies themes, occurrences and divergences in the ideas expressed by participants. Verbatim transcripts were reviewed by the research assistant who conducted the interviews, and the main author performed a first reading to segment the transcripts into units of meaning corresponding to the participants' expressed ideas (49). Each statement was assigned a specific meaning based on emerging themes and categories, which the research assistant validated for credibility (62). Categories were defined, and statements from the second phase were organized within these categories and subcategories. To ensure reliability in categorizing statements, a second research assistant participated in the analysis. Through comparison and discussion, an inter-judge agreement of 91% was reached, after which the primary coder continued the qualitative analysis independently. Thematic saturation was reached within seven interviews, as no new information regarding the satisfaction of the program emerged (63). However, to confirm saturation, three additional interviews were conducted, and all statements were validated through verbatim analysis.

## Results

This section presents the quantitative results of the questionnaires completed by participants together with qualitative feedback from parents' interviews regarding their satisfaction with the program.

### Quantitative

Participants reported a high level of intention to practice PA over the next three months, with a mean score of 6.94 (*SD* = 0.27). Accordingly, all participants (*n* = 11) also expressed a willingness (checked: *Yes*) to enroll again in the program *(*options*: Yes, No, I don't know)* in the next three months, confirming their intention towards PA engagement. Descriptive statistics for the intention to engage in PA over the next three months, comparative time points for the basic psychological needs and global self-esteem are presented in Table 2.

**Table 2:** Comparison time points for basic psychological needs and global self-esteem.

	*N*	Min	Max	*M*	*SD*
Autonomy T1	11	3.75	6.50	5.20	.93
Autonomy T2	11	5.00	7.00	6.01	.68
Autonomy T3	11	6.00	6.75	6.40	.23
Competence T1	11	4.00	6.60	5.23	.83
Competence T2	11	4.80	7.00	6.00	.73
Competence T3	11	5.20	7.00	6.36	.46
Relatedness T1	11	3.80	6.80	5.45	.99
Relatedness T2	11	5.60	7.00	6.38	.54
Relatedness T3	11	6.20	7.00	6.46	.30
Global self-esteem T1	11	3.00	3.70	3.23	.25
Global self-esteem T2	11	3.00	3.90	3.34	.28
Global self-esteem T3	11	3.00	4.00	3.56	.27
Intention T3	11	6.00	7.00	6.94	.27

Note: T1, at time 1 (first session); T2, at time 2 (7th session); T3, at time 3 (15th session); *M*, mean; Min,  minimum; Max, maximum; *SD*, standard deviation.

Results of the Friedman test point to significant differences over time for all three basic psychological needs as well as global self-esteem (see Table 3). Regarding competence need, the Friedman test revealed a significant difference over time, *χ*^2^(2, *N* = 11) = 11.74, *p* = 0.003. *Post hoc* analysis showed that competence satisfaction at T1 was significantly lower than at T2 (*Z* = −2.61, *p* = 0.009, *r* = 0.79) and T3 (*Z* = −2.68, *p* = 0.007, *r* = 0.81). However, there was no significant difference between T2 and T3 (*Z* = −1.37, *p* = 0.172, *r* = 0.41).

**Table 3:** Basic psychological needs and global self-esteem evolution.

Variable	Friedman test (*χ*^2^)	*p*-value	Post hoc comparisons	*Z*-value	*p*-value
Autonomy	13.54	.001	Time 1 < Time 2	−2.81	.005
			Time 1 < Time 3	−2.85	.004
			Time 2 = Time 3	−2.01	.044
Competence	11.74	.003	Time 1 < Time 2	−2.61	.009
			Time 1 < Time 3	−2.68	.007
			Time 2 = Time 3	−1.37	.172
Relatedness	7.95	.019	Time 1 < Time 2	−2.82	.005
			Time 1 = Time 3	−2.31	.021
			Time 2 = Time 3	−0.63	.529
Global self-esteem	12.60	.002	Time 1 = Time 2	−1.44	.150
			Time 1 < Time 3	−2.68	.007
			Time 2 < Time 3	−2.64	.008

Note: *Post hoc* comparisons used Wilcoxon signed-rank tests with a Bonferroni correction (significance level set at *p* < 0.017).

Autonomy need also showed a significant difference over time, *χ*^2^(2, *N* = 11) = 13.54, *p* = 0.01. The Wilcoxon signed rank test analysis revealed that autonomy satisfaction was significantly lower at T1 than at T2 (*Z* = −2.81, *p* = 0.005, *r* = 0.85) and T3 (*Z* = −2.85, *p* = 0.004, *r* = 0.86). However, autonomy satisfaction did not vary significantly across T2 and T3 (*Z* = −2.01, *p* = 0.044, *r* = 0.61).

In terms of relatedness need, the Friedman test indicated a significant difference over time, *χ*^2^(2, *N* = 11) = 7.95, *p* = 0.019. *Post hoc* analysis showed that relatedness at T1 was significantly lower than at T2 (*Z* = −2.82, *p* = 0.005, *r* = 0.85). The difference between T1 and T3 approached significance (*Z* = −2.31, *p* = 0.021, *r* = 0.70), suggesting a trend toward change. There was no significant difference between T2 and T3 (*Z* = −0.63, *p* = 0.529, *r* = 0.19).

Finally, with respect to global self-esteem, the Friedman test revealed a significant difference over time, *χ*^2^(2, *N* = 11) = 12.60, *p* = 0.002. *Post hoc* analysis indicated that global self-esteem at T2 was significantly lower than at T3 (*Z* = −2.64, *p* = 0.008, *r* = 0.80). Additionally, global self-esteem at T1 was significantly lower than at T3 (*Z* = −2.68, *p* = 0.007, *r* = 0.81), while no significant difference was found between T1 and T2 (*Z* = −1.44, *p* = 0.150, *r* = 0.43).

### Qualitative

Qualitative analyses identified two main categories assessing parental satisfaction with the program: benefits for the participants and acknowledgment of the program's value. These findings are based on interviews with parents (*n* = 10) whose children participated in the *BEB* program (See Supplementary file S4 for a thematic summary).

#### Benefits for participants

This category includes parents' reported and perceived benefits in all spheres of development (affective, social, physical, cognitive) for their child's participation in *BEB*. Some parents also described benefits that extended outside the program.

##### Affective

According to their parents, participants displayed notable improvements in emotional maturity, anger management and overall emotional regulation. These changes included greater emotional flexibility, improved self-reliance and fewer emotional outbursts. One parent shared the following:

I find he's matured, he's grown thanks to the Dek, and not just that he's grown, but also he's developed because of the Dek […] he used to have a lot of tantrums and now he does this a lot less since he started Bonheur en boule. (Parent 1).

This statement illustrates how parents perceived the program, through its activities, helped reduce emotional outbursts and fostered greater self-regulation. Parents also observed that children were better able to navigate their emotions, showing increased resilience in social interactions. As one parent stated, “*With time, things have really calmed down. Now, he's less rigid about certain things, like when he's talking with others at the Dek*” (Parent 9). Parents believe these emotional improvements were likely facilitated by the program's emphasis on teamwork, shared experiences and a supportive environment, which all encouraged children to express themselves and collaborate with others.

##### Social

Parents observed noticeable changes in their children's social behavior, particularly in terms of teamwork, communication with peers and the coach, and the development of friendships with children who shared similar experiences. One parent commented, “*In the beginning, he just wanted to play by himself, the ball was his, but after, he learned that no, there's the team, and then, we're here to have fun*” (Parent 1). This shows how parents felt the program helped their children understand the value of team spirit and collective play. In parents' opinion*, BEB* fostered a strong sense of belonging, enhancing team bonds and promoting cooperation and camaraderie. As one parent noted, “*There were newcomers we didn't know, and what we have to do then is include them*” (Parent 8). This highlights how parents perceived the program facilitated the integration of new children and strengthened friendships. Parents felt that the shared activities, including common dress-up, pre-session discussions, and use of a shared play area, further enhanced this sense of community, making children more eager to participate actively.

##### Physical

Here, parents underscored the progress in their child's abilities, noting significant increases in motor skills and dexterity. Specific observations highlight improvements in motor coordination, the ability to maneuver the ball more effectively, and faster movement across the field, along with more successful passes. One parent stated, “*He can throw passes, can do everything* […] *All his coordination has improved, so we see a very big improvement*” (Parent 4). What's more, several parents mention improvements in balance, agility and overall endurance. Another parent noted their child could now keep in pace with the game, showing greater confidence in his physical abilities. Still another commented, “*In the beginning, he did a half hour, 45 min, now he does a whole hour and we have trouble getting him to stop. That's where we see the improvement, he does the whole time*” (Parent 10). This improvement was also reflected in a stronger sense of body control and fewer physical hesitations during play. Furthermore, parents highlight a greater sense of mobility and responsiveness over time, attributing their child's improvements to consistent physical engagement in the program. One observed, “*He's much more mobile. In the beginning he walked along as the ball passed, he looked on. Now, he runs to get the ball*” (Parent 5).

##### Cognitive

Parents noted significant cognitive improvements in their children, particularly in areas such as understanding instructions, perception of time and surroundings, anticipation, attention and memory. These improvements were especially obvious in children's increasing awareness of the game, including their ability to anticipate plays and understand game strategies. According to one parent:

In the beginning he was passive […], We see he's a little more alert. He understands more about what's happening. He gradually came to understand more about passes, about shooting at the goal, etc. He shows cognitive improvement in line with his understanding of the game. (Parent 10).

This feedback illustrates how parents felt the program helped their child become more alert and better able to anticipate and engage with the game. Similarly, three parents (Parents 2, 6, and 9) also observed improvements in their children's ability to follow game-related instructions, maintain attention during play, and better remember key actions and strategies. One noted, “*As for the notion of time, he has a moderate to severe impairment, it's true, but he still manages to have a sense of time, and he knows something's about to happen*” (Parent 2). This highlights how parents described how their child improved their understanding of time and anticipation during the game.

##### Extended benefits

Parents also report various benefits extending beyond the program itself, including the acquisition of transferable skills, such as increased dexterity in other sports and enhancements in academic performance, particularly problem-solving skills. According to one person, “*Things were going well at school, so I think that helped him everywhere*” (Parent 4). Parents also noted improved behavior at home, including greater responsiveness and a better ability to follow instructions. One parent explained, “*Then, he gradually became less and less rigid at home too. He understands instructions and does what he's asked with less fuss*” (Parent 1). Additionally, the program contributed to improved relationships with other adults and the formation of social connections. One parent reported, “*We celebrated his birthday, we invited them, we went to play a big game of Laser Tag […] Then they switched to playing baseball too*” (Parent 8). This feedback underscores the program's possible positive impact on participants' social interactions and their ability to form new friendships and engage in new activities outside the program.

#### Acknowledgment of program's value

This category refers to parents' opinion of the program as a whole. It includes their points of view towards three subcategories: program details, intention of future participation and global appreciation.

##### Program details

Parents appreciate the program's flexibility, noting that it allows children to explore and play in a supportive, free environment. They also commend supervisors' ability to adapt to various situations, offering individual activities when necessary. This adaptability ensures that children facing difficulties are included and encouraged to progress at their own pace. As one parent shared, “*Then, when something's not working, they do other things, and there's no pressure eithe*r […] *they do shootouts, they try, they succeed, they adapt it so they succeed in the end*” (Parent 9). Additionally, the presence of caring and committed adults, particularly volunteers, is seen as a crucial element of the program. One parent enthusiastically noted, “*It was really great to see a group where everybody helped each other, where everybody congratulated each other and that we could all go there together. It's not just a competition. It's a family. A positive atmosphere*” (Parent 6). Parents also value the mixed group structure, where children with different disabilities come together. This diversity allows children to observe a range of needs and practice physical activity in an inclusive setting and, at the same time, reduces parental pressure. The result is a positive and non-competitive atmosphere, as the following person illustrates:

Myself, I find it's good because first of all, he can interact with other children who have other needs and therefore his need's not the only one, so that lets him see other aspects or persons who have different needs. Difference is beautiful in all its splendor. (Parent 4).

Parents feel this approach encourages mutual support, enhances development, and promotes a collaborative, positive environment for children.

##### Intention towards future participation

Every parent reported their child would continue with the program, reflecting a strong intention to remain involved. The motivations behind this intention varied, with parents citing factors such as fun, well-being, personal interest, social relationships and the overall enjoyment of the activities their child experienced. In the words of one parent:

So, I think that, really, he finds he's capable and he's having successes. I imagine these are the reasons for his enrollment. He asks us to play every season, so I think he likes it [..] He likes the atmosphere and the group. He always talks positively about them. (Parent 5).

This feedback underscores how the parents described the positive impact of the program on the child's experience and highlights their strong desire to continue. Another parent commented, “*We met other parents there, and we were hooked from the start, from the first session on, we kept enrolling him*” (Parent 8). This illustrates how the program triggered an initial enthusiasm in some parents that led to continued participation for their child. Similarly, another parent emphasized the benefits of the program, saying: “*First, because it's good for him and second, because I really love the program*” (Parent 6). These statements collectively demonstrate the various positive factors that drove parents' decision to keep their children enrolled in the program.

##### Global appreciation

Overall, parents who were interviewed expressed great appreciation for the program regarding both their children and themselves. Many parents highlighted the positive impact the program had on their children's emotional development and well-being. As an example, “*He's happy when he's participating, so you know, that's perfect, for sure. He develops a lot of good things*” (Parent 10). This reflection emphasizes the joy and personal growth their child experienced. Another parent reported, “*Just looking at [the children] makes me feel like going out there to play. You can feel the hockey sticks and the balls, and you feel like playing, that feeling's contagious too*” (Parent 5). This underscores the contagious enthusiasm and excitement the program generates for some parents. Parents also noted the broader impact of the program on family dynamics, as the following shows:

Thanks so much, it has such an impact, you know. We're speaking for the child here, but really, it has a huge impact, plus more for the parents because it strengthens the bond between the parent, the child […]. They have fun, they're happy, they laugh. It's their activity. (Parent 7).

This highlights how the program could have strengthened the parent-child bond and brought joy to both. Additionally, another parent indicated that the program offered some positive relief to children facing significant challenges:

As for our children with big challenges, well, there are negative challenges at school, you know, because it's not the same context […], there, they can experience something positive but it also leads to a good outlook. (Parent 7).

This suggests that, despite challenges in other areas of life, the program may offer a positive experience that helps children focus on their strengths and develop a more positive outlook.

Overall, while all the parental reports seem promising, it is important to note that the sample was small (*n* = 10 parents), which may limit the generalizability of the results.

## Discussion

This study aimed to assess how an adapted PA program (*BEB*) influences participants' basic psychological needs, global self-esteem and intention to engage in PA over a three-month period together with parents' satisfaction with the program. Results indicate that the satisfaction of participants' basic psychological needs (autonomy, competence and relatedness) as well as global self-esteem increased over time. These findings suggest that a PA program based on SDT for youth with disabilities may represent a promising approach. Moreover, the qualitative analyses highlighted multiple benefits of the *BEB* program in cognitive, affective, physical and social domains, contributing to the high satisfaction levels reported by parents.

### Evolution of participants' basic psychological needs and global self-esteem

As regards the first objective of this study, i.e., examination of the basic psychological needs and global self-esteem, participants report an overall increase in satisfaction concerning autonomy, competence and relatedness as well as global self-esteem over time. Notably, the significant increase found between T1 and T2 across all basic psychological needs suggests that the program successfully met participants' needs early on.

The growing satisfaction of the competence need among participants is likely explained by the instructors' consistent encouragement during each session, which reinforced a sense of achievement. This motivational strategy supports the existing literature suggesting that positive feedback and achievable goals are critical for competence development among youth with disabilities as regards PA (4, 64). In line with this finding, parents mention that the sessions included drills and practices that respected children's limits, potentially fostering their sense of competence.

The increased satisfaction of the autonomy need (mainly from T1 to T2) among participants may relate to the freedom, choices and sense of control they experienced when participating in the program's activities. According to previous studies, respecting individuals' needs and paces while allowing them to make their own decisions about their progress is crucial for satisfying the need for autonomy (28, 65, 66). Likewise, research suggests that adopting a more supportive style of guidance and fostering a freer environment in PA is key to promoting autonomy among youth with disabilities (67). Thus, the instructor's approach of intervening to lead and guide children only when necessary, while encouraging them to play as they wish and make their own choices, likely contributed to their sense of autonomy. This explanation is well supported by parents who report that the program's flexibility allowed their children to choose both their activities and the timing of their participation.

Finally, the improved satisfaction of the relatedness need among participants may be explained by factors such as teamwork, inclusion, group play, participants' mutual support, parents, and the instructor as well as discussions by the instructor that extended to events beyond the program. As previous research suggests, supportive social environments in PA characterized by fair and equitable participation, a sense of belonging through teamwork and opportunities for interdependence facilitate and encourage relatedness for youth with disabilities (68, 69). Parents also report that the instructor's presence promoted inclusivity in all group activities and play, fostering a sense of belonging and connection. This approach is consistent with research stressing the importance of social integration for greater relatedness (70).

However, although a significant increase was observed between the start and middle of the season (i.e., between T1 and T2) for each basic psychological need, there was no significant rise between T2 and T3. This could be because mean scores were already high at T2 for all three basic psychological needs (*M* ≥ 6.00), suggesting that after seven sessions, participants already felt a sense of autonomy, competence and relatedness that was sustained until the end of the season. Nevertheless, the difference between the relatedness need measured at T1 and T3 and measured at T2 and T3 was not significant. We believe this is because over half the participants (*n* = 7) had taken part in the program in previous years, giving them a high sense of relatedness from the start (*M* = 5.91). The significant change from T1 to T2 could, therefore, be attributed to new participants' affiliation with former participants. Still, although the difference between T1 and T3 was not significant, it indicates a clear trend towards increased satisfaction of the relatedness need over time (*M* = 6.46; *p* = 0.021).

As for global self-esteem, participants did not report improvement until later in the program (i.e., between T2 and T3). Hence, results suggest participants may take more time to develop global self-esteem and realize their potential. This finding agrees with previous studies, such as that of Scarpa (71), stipulating that PA tends to positively impact self-esteem. However, the finding of the current study stresses that perseverance and support are essential to build self-esteem, as its process of development appears to be more gradual than that of other psychological needs. Implementing longer programs, therefore, could potentially lead to a more significant and sustained increase in global self-esteem over time for youth with disabilities.

### Intention to pursue *BEB* program and physical activity

Concerning the second objective, measure participants' intention to pursue the program and practice PA afterwards, all participants expressed a desire to pursue the program and demonstrated the intention to engage in PA during the following three months. This is an indicator of PL, as it reflects their confidence and competence toward PA participation with a view to maintaining an active lifestyle. Indeed, previous research suggests that sustained motivation and participation in PA are crucial elements of PL and are essential for leading a physically active life (19, 38, 72, 73). Therefore, it is important to note that all participants in the program from the fall season continued in the winter season. However, we did not specifically measure PA engagement outside the program, which could lead to uncertainties regarding true continuation in PA participation in the near future.

### Parents' satisfaction and benefits of the program

Regarding our third objective, i.e., describe parents' satisfaction with *BEB*, parents report notable improvements in their children's understanding of instructions, perception of time, attention span and memory retention, emphasizing the cognitive gains facilitated by participation. These cognitive gains suggest that the program significantly enhanced participants' ability to process information and engage with the game on a deeper level. For example, parents tend to stress the importance of cognitive skills like inhibition and memory, largely because they can observe them at home and be offered feedback from the school. Thus, participation appears to develop a sense of focus, understanding and reflection in children. Detailed explanations about the game and drills along with an emphasis on autonomy, which is supported by quantitative results showing an increase in autonomy need satisfaction over time, allow children to pause to reflect and adapt to various situations with the support of their instructors. This structured yet flexible approach seems to foster cognitive development indirectly. The assessment of cognitive improvements in PA can indeed be challenging, as it requires specific materials, knowledge and measurement tools (74–76).

Parents also report enhanced emotional regulation and reduced instances of emotional outbursts in their children, underscoring the program's impact on their affective development. The reason is most likely the program's structure, which offers participants the freedom to play and express themselves upon entering the field, while making instructors available to provide support as needed. As well, the diversity within the groups, in terms of both age and disability, fosters a nurturing environment. For example, older or more experienced participants frequently act as mentors by helping newer or younger participants. This dynamic encourages newer participants to focus on the game and observe how more advanced players manage their emotions and behavior. This peer-to-peer interaction not only promotes a caring atmosphere, but also enhances the overall emotional and social growth of all participants. To our knowledge, no literature to date has addressed peer-to-peer mentoring between people with disabilities in PA settings. However, previous research underscores how peer-to-peer support in sports programs enhances social inclusion and empowerment for individuals with disabilities, fostering a supportive and nurturing environment (77, 78). Although this aspect was not a component of the *BEB* program, the emergence of this dynamic highlights the potential of peer-to-peer support as a mechanism for promoting relatedness and engagement in PA program settings. Given its potential to foster social and affective development, peer-to-peer support might represent a relevant strategy to facilitate inclusion and should be explored in future studies within adapted PA interventions.

Similarly, the development of social bonds, improved relationships with peers and a heightened sense of camaraderie among participants highlight the program's social benefits. *BEB* emerges as a beacon of social inclusion in community settings, promoting a supportive and accepting environment where children with diverse disabilities can thrive. As such, the program not only satisfies the need for relatedness, but also allows parents to observe friendships being formed through the interaction of multiple individuals with different disabilities. This is important given that these individuals often have fewer relationships than their non-disabled peers (22, 23). Furthermore, this collaboration between participants has proved to be an advantage in unforeseen ways. Specifically, according to their parents, children have become more attuned to the needs of others and actively assist them whenever possible. This is supported by quantitative results, which reveal an increase in relatedness need satisfaction over time. Indeed, one of the key points of group-based PA is to promote PA participation via sports interaction, which often yields multiple social benefits. Some studies show similar findings, insofar as PA participation serves to foster a sense of acceptance by creating opportunities for friendship and encouraging peer-to-peer interactions (21, 79). Children and adolescents in these programs report smoother social interactions (79) and improved social communication skills because team sports encourage players to support each other (80). Thus, parents report that participation in group-based activity programs like *BEB* appears to improve interpersonal and social skills (81).

Overall, examination of the qualitative data uncovers insights into the potential long-term implications of *BEB* for children's development and the sustainability of its positive outcomes. In terms of sustainability, parents point to the program's adaptability, flexibility and emphasis on inclusivity as the key reasons for its longevity and resilience. Said adaptability and flexibility inform both parents' trust and young people's confidence, as the activities are implemented in a safe and friendly environment. In parents' view, these components are important because they address children's needs as well as parents' concerns and uncertainties regarding their children's well-being when participating in the program. There's no doubt, as previous research shows, that parents of youth with disabilities often fear their child will experience difficulties, failures, injury or the teasing of other children (15, 82). This can limit opportunities for these young people because parents seek to prevent such problems and consequently reduce the occasions for their child to engage in activities (82).

Alternatively, and as mentioned in the interviews, parents who trust the program tend to keep their children enrolled for a longer period, thereby promoting long-term benefits. In fact, parents report that certain long-time participants continue to demonstrate its benefits. Similarly, previous research shows that parents' perceived support and needs fulfillment also play a crucial role in PA participation (83). Parents' interview responses focus, notably, on satisfaction of the three basic psychological needs and the development of PL, even though the interview template did not specifically include these topics (See Supplementary file S3). This needs fulfillment supported their decision to have their children participate in the following season. Previous studies (84, 85) maintain that parents' support and perceptions of PA are immensely important, as they often act as facilitators or obstacles to their children's participation. Their positive perception of the *BEB* program not only makes it more likely their children will continue participating, but also encourages them to explore additional PA opportunities.

### Comparison with adapted PA interventions in other settings

Some systematic reviews have shown the effectiveness of school-based, therapy-based and clinical-based adapted PA interventions in improving motor skills, fitness or social development (86, 87). These settings often emphasize on structured and controlled environments to ensure participation, guided by trained individuals. Some programs grounded in SDT or PL, mainly in school-based settings, have also highlighted the importance of satisfying the three basic psychological needs to enhance motivation and foster key components of PL to sustain engagement in PA (25, 88–90). In comparison, the *BEB* program's flexible and inclusive approach may foster greater peer-to-peer interaction, autonomy and motivation, particularly because participation is voluntary and less externally regulated. Additionally, since the program operates in a real-life setting, the outcomes gained from the program may be perceived differently by participants, as it directly engages their capacity to take action outside controlled environments such as schools and clinical settings. Therefore, this context may enhance their self-esteem and motivation in a unique way, as accomplishing goals or actions in everyday life can feel more meaningful and empowering compared with controlled settings. Overall, while more research is still needed on adapted PA programs, these findings highlight how community-based program like *BEB* could complement school or clinical settings, pointing to several practical implications for future program development.

### Practical implications

In general, appreciation for the program and the intention to engage in PA over the coming three months show that a PA program focused on satisfying the needs for competence, autonomy and relatedness provides a strong framework for interventions targeting individuals with various disabilities. Consistent with previous studies, participants' intention to pursue PA is often associated with perceived autonomy, competence and relatedness, whose fulfillment increases engagement and PA participation (91).

Accordingly, a particular element fostering a sense of relatedness is the framework formed by the program's composition and values (see TIDieR; Supplementary file S1), which encourages a supportive and inclusive environment. Multiple parents report the program felt authentic and made their child feel part of a larger organization, of a team where each person had a role to play and was involved every step of the way. Thus, promoting environments where each person feels part of the group, and which promote support, safety and well-being through positive interactions, is essential for programs aimed at building strong connections and a sense of belonging. In addition, programs should consider holding outside events including tournaments, official competitions, group dinners and recreational activities to strengthen a sense of community among participants.

Another key factor is the program's flexibility, which was consistently emphasized by parents and directly linked to the autonomy need. Thus, it's essential to maintain flexibility when structuring programs and meet participants' needs by offering choices at every stage and remaining open to suggestions and ideas from both parents and participants. Even when the role played is a minimal one, ensuring that participants' and parents' voices are heard and taken into account is crucial for promoting autonomy and engagement.

In terms of the competence need, an important aspect is offering participants sufficient space to discover new skills, explore new methods, try new movements and become familiar with the equipment and environment. The fact the instructor intervenes only when a participant asks for help, requires a demonstration, or encounters significant difficulty is essential to support and maintain this approach. This process allows participants to act and persevere on their own, fosters effort and promotes success by adapting certain parts of exercises or gameplay to individual abilities. Moreover, the use of specialized materials conduces to the discovery of new competencies and skills. Programs should therefore introduce a greater diversity of activities, equipment, and challenges to further support skill development and encourage creativity among participants by allowing them to explore at their own pace, even when they don't immediately succeed.

Parents say that their main reasons for continuing with the *BEB* program are their child's interest and enjoyment. Certainly, the importance of choosing a preferred PA to have fun while being motivated and focused promotes enjoyment and involvement (92). As well, the interests of young people with disabilities must be emphasized in programs to support their motivation, as this aligns with STD and targeted benefits (28, 30).

Additionally, a study conducted by Shields et al. (93) demonstrates the effectiveness of pairing children with disabilities with non-disabled children. The current study expands on this method by pairing more experienced players with less experienced ones, regardless of their differences. In fact, parents mention that the involvement of participants with multiple disabilities was a new and enriching experience for both their child and themselves. It helped them realize it's possible for those with limitations to engage in PA with the support of adapted measures and a dedicated staff. Moreover, the diversity of abilities among participants encouraged children to develop their own approaches or techniques by observing and learning from other children using different methods. Similarly, a study conducted by Willis et al. (94), argues that group-based interventions that foster a supportive atmosphere, encourage social connections, provide role models, and adapt activities to children's abilities are crucial components for meaningful participation.

On another note, parents could easily observe cognitive improvements in their children, indicating their importance in the evaluation of cognitive development. Parents should thus be considered more often, as the assessment of cognitive components requires more materials, knowledge, and measurement tools (74–76).

Finally, more steps must be taken to improve recruitment; an example is by reaching out to more girls. As parents report in the interviews, most participants were drawn to the program through networking. Leveraging this method could be an effective strategy to promote the inclusion of girls. Additionally, targeting outreach campaigns, collaboration with schools, and partnerships with community organizations could further enhance recruitment efforts and ensure greater participation.

### Study limitations and directions for future research

This study's key strength involves implementing a program that fosters the basic psychological needs and global self-esteem of youth with different disabilities by combining the SDT framework with PL to promote PA participation**.** However, it includes several limitations that are addressed and grouped into three themes: sample-related, methodological, and generalizability concerns.

#### Sample-related limitations

First, the sample size was small, as many children were unable to participate owing to comprehension difficulties. As well, one participant was obliged to drop out for personal reasons, resulting in some missing data. Future studies should therefore use larger samples over longer periods to improve the reliability and generalizability of results while minimizing the impact of participant dropout, especially in view of the challenges faced by individuals with disabilities. Second, all participants were boys, possibly because of the higher prevalence of certain disabilities (e.g., ASD) (51) found in males. This gender imbalance, favoring males, may limit the generalizability of the findings, as no female participants were included in the current study. In future studies, more inclusive recruitment strategies should be implemented, such as targeted outreach, school collaborations, parents networking and promotion through local community centers, in order to promote greater gender diversity and increase participation.

#### Methodological limitations

Third, the current study did not specifically document or measure the effects of peer-to-peer mentoring and nurturing, which likely contributes to the satisfaction of the basic psychological needs. Further research is needed regarding peer-to-peer mentoring and the nurturing process in PA settings among youth with disabilities as this may be an interesting way to enhance relatedness and PA participation. Fourth, this study was based entirely on self-report questionnaires, which can introduce bias, inaccuracies and inconsistencies because of their reliance on participants' subjective perceptions. To limit bias related to self-reported measures, particularly regarding the basic psychological needs and intentions, future studies could incorporate objective assessments (e.g., physical tests, accelerometers, performance metrics or observational data) and third-party observations by trained observers, ensuring a more accurate representation of participants' experiences. Likewise, the measures of participants' intentions did not include direct assessments of behaviors that would confirm these intentions. More research is therefore needed to evaluate PA participation in the months following the program to determine whether intentions translate into sustained behavioral changes and to assess the program's long-term impact on participants' PA levels.

#### Generalizability limitations

Fifth, the program did not include certain disabilities (e.g., visual impairments, hearing impairments), limiting the generalizability of the outcomes. Future research should consequently explore heterogeneous group settings involving less common disability types (e.g., Fragile X syndrome), as this approach offers more realistic scenarios and potential strategies for implementation in community environments to promote inclusivity. The sixth and last limitation concerns the absence of previous PA experience measurement as a confounding variable, along with the lack of a control group for comparison, which could hinder the validity of this study and warrants caution when interpreting the results as evidence of a causal relationship. Future research should therefore address these issues by including a control group and considering participants' previous PA experiences to enhance the study's validity.

## Conclusion

Most studies examining PA programs focus on assessing the effectiveness or outcomes of interventions while often neglecting critical factors such as feasibility, reproducibility, and validity for implementation in community settings or real-world applications (39). However, *BEB* shows the possibility of implementing an adapted, group-based PA program using a framework grounded in SDT to promote PL behaviors and benefits through cognitive, affective, physical, and social development for youth with diverse disabilities. A key indicator of PL is fostering lifelong engagement in PA (19). The development of PL not only enhances participation in PA, but also supports further advancement of PL itself (38). In this study, these effects are seen in the improvements across multiple domains (cognitive, affective, physical, social), equipping children with valuable tools to navigate future challenges and opportunities, such as engaging in other PA programs. While these results are encouraging, they should be interpreted with caution given the small sample size and the lack of control group. Further research using experimental designs, such as controlled trials and longitudinal studies, is needed to validate the program's potential for broader use and sustainability. Furthermore, parents' expressed intentions for their children to continue in the program underscore its perceived value and feasibility, but these perceptions still require further examination through behavioral data.

## Data Availability

The raw data supporting the conclusions of this article will be made available by the authors, without undue reservation.
