# Supplementary material for: Bonheur en boule: an adapted group-based physical activity program for youth with disabilities
Source: Front Sports Act Living. 2025 Jul 31;7:1580697. doi: 10.3389/fspor.2025.1580697 (PMC12352332; doi:10.3389/fspor.2025.1580697)
Supplement: Supplementary file 1 [file Supplementaryfile1.docx]

**Supplementary file 1.**

Description of the *Bonheur en boule* program (TIDieR)

| Brief name | | Bonheur en boule (*BEB*) | | | | | |
| --- | --- | --- | --- | --- | --- | --- | --- |
| Why | | Youth with disabilities face multiple challenges in accessing and participating in physical activity programs that are beneficial for their development and well-being. The *Bonheur en Boule* dek hokey (ball hockey) program was created to provide an opportunity for youth with diverse disabilities to engage in an adapted activity program tailored to their specific needs, promoting their inclusivity and participation.  Security, well-being, and enjoyment are the foundation and values of the program.  Theoretical underpinning: The program was developed based on Self-Determination Theory (SDT) as a theoretical framework, while also promoting physical literacy, as outlined in the introduction of the paper.  For a clearer understanding of the program's fundamentals, visit the French version of the guide at: https://bonheurenboule.ca/index.php/guide-bonheur-en-boule/. | | | | | |
|  |  | Motivational strategies implemented in the program to sustain basic psychological needs | | | | | |
|  |  | Autonomy | | Competence | | Relatedness | |
|  |  | • Players are given the opportunity to choose between different exercises or practice the skills they feel most motivated to work on.  • Instructors encourage players to take control of their learning process, allowing them to experiment with movements and execute them in a way that feels right.  • Players are free to explore and move freely around the playground. | | • Instructors consistently highlight positive progress and provide feedback to support participants' development.  • When instructors observe that a participant is improving, they introduce appropriately challenging tasks that promote further growth and success.  • Instructors adjust exercises to ensure that every participant can fully complete each drill at their own level of ability. | | • Instructors organize team drills and activities to foster collaboration and strengthen mutual connections among participants.  • Instructors engage in conversations beyond the program to build connection and create a sense of community.  • Instructors spend individual time with each participant to ensure they feel included and valued, showing genuine care for their unique needs. | |
| What | Materials | For instructors: The training involved informational materials necessary to hold meetings and PowerPoint presentations that addressed both theoretical and practical concepts of inclusivity and adaptation, including specific considerations for all disabilities represented in the program.  For participants: The session involved physical materials which considerably vary for each season according to the interest and creativity of the instructors. Here are some of the items used in the current study (required equipment to play is not mentioned here):   - Typical hockey balls (orange) - Tennis balls - Multiple colored cones - Mini flashlights - Balloons - Ropes - Colored tapes - Puck crates - Hockey goal target   For a quick view of the required equipment visit: https://bonheurenboule.ca | | | | | |
|  | Procedures | Free Play (10 mins) | Training (15 mins) | | Game (25 mins) | | End-Game (10 mins) |
|  |  | The free-play period is a free time where the players are on the field with different types of balls and special material, which they can use as they want. They can dribble, pass the ball with others, shoot to the net, shoot on the goalie, or talk with the instructors and other players. | The training time is where the instructors are proposing different types of exercises to the participants, which they can choose from or continue free play. Each exercise is explained and demonstrated by the main instructor with the help of the staff. These exercises consist of multiple skills and knowledge about the game (e.g., controlling the ball, passing, placements, shootings, rules, etc.). | | During the game time, the players decide their own team with the help of the instructors. While playing, the instructors help the players with different skills, placement, tips, and tricks. Sometimes, the instructor will ask the referee (another instructor) to pause the game to explain certain aspects of the game or breaks. | | The endgame includes a shootout session, which is a one-on-one confrontation between the goalie and the player. The shootout ends when all players scores one or two goals. All players usually do two to three shout-outs. Every shootout ends when the player has successfully scored a goal. |
| Who provided | | The main author (also the main instructor of the program), who has 10+ years of experience in adapted physical activity, a degree in psychology, as well as an expertise in both Self-Determination Theory (SDT) and Physical literacy (PL), lead all sessions of the program.  Staff members (instructors), who have a background or an expertise among youth with disabilities and hold qualifications in various fields of intervention (e.g., psychology, psychoeducation, social work). All staff received instruction regarding the procedure of the program and its main goal before every season, through staff meetings representing 10 hours of formation per season delivered by the main author with the help of two other professionals (educational psychologists). | | | | | |
| How | | Each session is conducted in a group setting, but instructors also provide one-on-one support when needed, such as demonstrating specific movements, managing crises, engaging in free discussions, assisting with equipment, and addressing other individual requests.  To ensure security and enjoyment for everyone, participants are grouped based on their age, needs and prior experience in physical activity or dek hockey. At the beginning of each season, the main instructor holds meetings to assess the characteristics of the participants with the help of the other instructors. This is followed by an observation session during the program's first day, conducted by all instructors, which leads to group creation. A subsequent meeting is held to finalize the groups, which are then validated based on the participant's sense of well-being (the participant has is word to say) and feedback from their parents, aligned with the instructors' recommendations.  All participants of the programs are divided between two groups: group 1 (usually 5-12 years old; *n* = 7) or group 2 (usually 13-24 years old; *n* = 8). | | | | | |
| Where | | Participants attended a community center each week, where the program takes place. | | | | | |
| When and how much | | The program is held once per week (every Sunday at 10:00 am UTC-5) for one hour on a period of 15 weeks. Intensity and duration varied according to each participant's energy levels, motivation, and willingness to continue. | | | | | |
| Tailoring | | The program is designed to be responsive to the needs of all participants. Each exercise and gameplay activity are adapted in intensity and structure to align with participants' abilities. These adaptations may include using specialized equipment, such as a different type of ball or brightly colored cones, to simplify the task. Alternatively, adjustments may involve modifying the activity itself, such as slowing down the pace, breaking the exercise into smaller, more manageable steps, or having participants follow the instructor’s demonstration closely. See supplementary file 2 for detailed adaptions through each phase of the program (arrival to end). | | | | | |
| Modifications | | The intervention went as planned. | | | | | |
| How well | Planned | The fidelity of the program is assured by the main instructor, who evaluated the program through a one-year pilot phase followed by an assessment period of two years to make sure each component of the program was in accordance with the main objective.  Afterwards, over a three-year period, the adaptation of the program was achieved by aligning it with the evolving needs of new participants each year. As these needs emerged, the program incorporated insights from SDT and PL to ensure the program remained responsive and effective. Strategies to maintain and improve fidelity included training protocols for instructors, regular meeting sessions, and the use of participants and parents feedback to ensure consistency in program delivery. This ongoing process allowed the program to better address the unique needs of each participant, while integrating theoretical frameworks to enhance the program's overall impact.  Overall, the program served over 100 participants over the spawn of seven years. Still to this day some participants remain in the program. | | | | | |
|  | Actual | The fidelity of the study’s implementation was ensured by the main author, who served as the primary instructor of the program, a role he has led for more than seven years | | | | | |
