# Supplementary material for: Bonheur en boule: an adapted group-based physical activity program for youth with disabilities
Source: Front Sports Act Living. 2025 Jul 31;7:1580697. doi: 10.3389/fspor.2025.1580697 (PMC12352332; doi:10.3389/fspor.2025.1580697)
Supplement: Supplementary file 2 [file Supplementaryfile2.docx]

**Supplementary file 2.**

| **Arrival** | **Group setting and environment** | **Free play** | **Training** | **Game** | **End-game cooldown** | **After-game** | **Crisis** | **Staff** |
| --- | --- | --- | --- | --- | --- | --- | --- | --- |
| Upon arrival, each player is greeted and directed to a designated calm room where chairs are arranged in a circle to encourage communication and foster a sense of relatedness. This room serves as a gathering space for players to put on their equipment and for the instructors to provide any necessary explanations. This room also serves to establish routines and promote inclusivity, ensuring that no one is left behind. | At the start of the season (week 1), all the participants are attending the same group. This first attendance serves for the instructors to evaluate the youth abilities and needs regarding their disabilities and age. During this stage, all players play against the instructors and are randomly distributed in groups of 4. Each group play against the instructors during the respective 60 minutes length. They are further assigned to group 1 or group 2, depending on the evaluation of the instructors. It is important to note that this evaluation is mainly done to assure security and to avoid significant disparity in participants as the program focus on well-being and fun. Otherwise, when groups are completed, groups are constituted of 10-15 participants with an instructor to child ratio of 1:2. | The primary aim of free time is to provide young individuals with the opportunity to familiarize themselves with the surroundings, socialize with others, fostering autonomy and exploration while others are arriving. It also serves as a preparing period for instructors to set up the exercises. | To avoid waiting times, the group is often divided in half as it facilitates explanation and approaching the players in a more personal manner. Trainings are designed to challenge and respect the capacity of the players. While doing the exercise, one instructor follows the player to make sure there is no complications during the exercise. Instructors provide additional support when needed. | During the game, we make sure that the teams are well-balanced by making switches or adding instructor(s) into a specific team, if necessary. Therefore, staff in charge make sure that all players are in touch with the ball and feel included throughout the game (e.g., they can pass them the ball once they have it or even use another one). | The shout-out period is an important part of the program as every player is assured to score a goal at the end of the session. Therefore, this part is mainly used to promote a sense of success for all players. (One out of the two goalies is an instructor). | After each session, the group reunite into the same designated room and are free to socialize while removing their equipment. This period serves as a social moment for youths and parents. This time is also dedicated to making announcements about upcoming activities or events. | Whenever a crisis occurs, the instructors ask the referee to pause the game and every player go into a break time. This break time let the instructor enough time to act and apply the intervention accordingly to the situation. If the situation persists, the associated players are accompanied by an instructor out of the playing field to talk about the situation and let the other players continue the game. Otherwise, parents are often near if anything else requires their attention or their presence through more specific situation. | At every start of each season, the main instructor holds meetings to observe the different characteristics of the participants. These meetings serve as an analyst point for all staff members to propose adaption to respond accordingly to any special needs of the participants. The varied backgrounds and collective experience of the staff facilitate a more accessible approach to specific types of disabilities, enabling effective adaptation to special needs. |

*Adaptation details of the program*
