# Supplementary material for: Bonheur en boule: an adapted group-based physical activity program for youth with disabilities
Source: Front Sports Act Living. 2025 Jul 31;7:1580697. doi: 10.3389/fspor.2025.1580697 (PMC12352332; doi:10.3389/fspor.2025.1580697)

**Supplementary file 3.**

**Interview Framework for the *Bonheur en Boule* Program**

The objective of this interview framework is to assess parents' satisfaction regarding their child's participation in the *Bonheur en Boule* program.

1. How long has your child been participating in the *Bonheur en Boule* program? And why did you decide to enroll your child?
2. How would you evaluate your experience with the program so far?
3. Does the program meet your child’s needs? Why or why not?
4. Does the program meet your expectations? Why or why not?
5. What aspects of this program have you found most beneficial for your child and/or for yourself?
6. Are there any aspects of the program you would like to see improved?
7. Have you noticed any changes or improvements in your child since participating in the program? If yes, what are they? If not, why do you think that is?
8. Do you have any suggestions to make this program even more effective or better suited to your child’s needs?
9. Would you recommend this program to others? Why or why not?


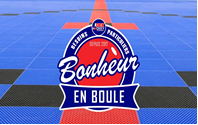

Supplement: Supplementary file 3 [file Supplementaryfile3.docx]
