# Supplementary material for: Bonheur en boule: an adapted group-based physical activity program for youth with disabilities
Source: Front Sports Act Living. 2025 Jul 31;7:1580697. doi: 10.3389/fspor.2025.1580697 (PMC12352332; doi:10.3389/fspor.2025.1580697)
Supplement: Supplementary file 4 [file Supplementaryfile4.docx]

**Supplementary file 4. Thematic table.**

**Note.** All descriptions and quotes have been freely translated from the original French interviews. As such, slight differences in tone or phrasing may occur compared to the original wording.

| **Main Theme** | **Subtheme** | **Description** | **Additional quotes** |
| --- | --- | --- | --- |
| Benefits for the participants | Affective | Affective benefits experienced by the children are described as increased self-awareness, maturity, autonomy and the emergence of positive emotions, thoughts, behavioral traits, or feelings associated with participation in the *Bonheur en boule* program, as perceived by parents. | *“It really helped him manage his anger. Especially, when he was dealing with small frustrations.” (Parent 7).* |
|  | Social | Social benefits experienced by the children relate to social development, including social behaviors, communication, sense of belonging and all other positive aspects of group interaction following their participation in the *Bonheur en boule* program, as perceived by parents. | *“There’s another little player on the team. When he sees him arrive, he goes to talk to him a bit more. He’s starting to talk more with his friends than he did three years ago.” (Parent 4)* |
|  | Physical | Physical benefits experienced by the children are presented as improvements in motor development, including coordination, dexterity, motor skills and other physical abilities following their participation in *the Bonheur en boule* program, as perceived by the parents. | *“He plays once a week and now he handles the ball really well. He can, you know, spin around with it.” (Parent 9)* |
|  | Cognitive | Cognitive benefits experienced by the children are presented as improvements in perception, attention, memory, reasoning, problem-solving and other thinking-related abilities following their participation in the *Bonheur en boule program*, as perceived by the parents. | *“When we tell him things, he listens really well and you know, he understands the first time. We've really seen improvement in that area.” (Parent 10)* |
|  | Extended benefits | Extended benefits experienced by the children are described as positive effects observed outside the program setting but perceived by parents as resulting from their participation in the *Bonheur en boule* program. These benefits may manifest across different developmental domains and in various environments such as home, school, or other social contexts. | *“His teacher noticed that he was much more patient during sharing time and his temperament seemed calmer lately. We’re really happy that his school environment has improved.” (Parent 1)* |
| Acknowledgement of program’s value | Program details | Program details are presented as parents’ positive or negative opinions regarding various aspects of the program, such as its structure, procedures, frequency, participant selection criteria, staff members, duration, content, group dynamics, methods and other related characteristics. | *“And like I said earlier, there’s real adaptation. There aren’t many places where you can do sports and actually feel that. It’s not just one coach for 30 kids. They’re really well supported and it’s honestly wonderful. There should be more of that. More adapted sports for kids who otherwise can’t participate.” (Parent 1)* |
|  | Intention towards future participation | Intention towards future participation are presented as the motivations, reasons, or conditions shared by parents that explain whether or not their child continued participation in the *Bonheur en boule* program. | *“He always wants to keep going. When the season ends, we ask him and every year he says yes. I think he really likes the group and the team spirit the program brings.” (Parent 3)* |
|  | Global appreciation | Global appreciation are presented as parents’ positive or negative opinions regarding their overall experience with the *Bonheur en boule program*, including its impact on their child and family as a whole. | *“At first, I was a bit hesitant, but he was well supported and it was great to see his progress. It’s truly a wonderful program. Personally, I’d give it a 10 out of 10.” (Parent 2)* |
